# What Thinking Like a Bioethicist Can Bring to Dementia Research

**DOI:** 10.1093/geroni/igaa057.2026

**Published:** 2020-12-16

**Authors:** Nancy Berlinger, Kate de Medeiros, Laura Girling

**Affiliations:** 1 The Hastings Center, Garrison, New York, United States; 2 Miami University, Oxford, Ohio, United States; 3 University of Maryland, Baltimore County, Baltimore, Maryland, United States

## Abstract

Bioethics is an interdisciplinary field that uses critical and empirical tools to explore and make recommendations concerning uncertainty about duties to others, including socially marginalized populations. In the context of social science or biomedical research involving people living alone with dementia, practical challenges in conducting research with capacity-impaired participants have ethical dimensions concerning informed consent and other aspects of research conduct. The underrepresentation in dementia research of the voices and perspectives of people living at home with dementia raises normative questions. Using data from a recent National Institute on Aging bioethics supplemental grant, this paper explores how thinking like a bioethicist can strengthen gerontological research. This paper examines areas such as precarity of housing, poverty and social interactions from a bioethicist’s critical analysis/perspective and provides a framework for others to apply to their own research.

